# Disasters, Gender, and HIV Infection: The Impact of the 2010 Haiti Earthquake

**DOI:** 10.3390/ijerph18137198

**Published:** 2021-07-05

**Authors:** Mar Llorente-Marrón, Yolanda Fontanil-Gómez, Montserrat Díaz-Fernández, Patricia Solís García

**Affiliations:** 1 Quantitative Economics Department, University of Oviedo, 33006 Oviedo, Spain; mdiaz@uniovi.es; 2 Psychology Department, University of Oviedo, 33003 Oviedo, Spain; fontanil@uniovi.es (Y.F.-G.); solispatricia@uniovi.es (P.S.G.)

**Keywords:** disasters, gender, determinants of HIV seropositivity, difference-in-difference model, gender gap

## Abstract

Although disasters threaten all people who experience them, they do not affect all members of society in the same way. Its effects are not solely restricted to the economic sphere; they also affect the physical and mental health of those who suffer from them, having a particular impact on women and limiting their life chances. The aim of this study was to examine the impact the 2010 Haiti earthquake had on the seropositivity of female survivors. Method: Using data from the Demographic and Health Survey, this study examines the impact of the 2010 Haiti earthquake on gender relations associated with the probability of being HIV positive through the differences-in-differences strategy. Results: A differential of four percentage points is observed in the probability of HIV seropositivity between men and women, favoring men. Additionally, it is observed that the probability of seropositivity intensifies when the cohabitation household is headed by a woman. Conclusion: Disasters are not indifferent to the gender of the people affected. In the second decade of the 21st century, the conclusions obtained show, once again, the need for incorporating the gender perspective into the management of natural hazards in the field of health. This is the case of the differential exposure to HIV after the earthquake in Haiti.

## 1. Introduction

### 1.1. Disasters: A Gender Issue

A quarter of a century ago, Fothergill (1996) [1] denounced the negligence during the investigation of the different consequences of disasters and their dependence on the gender of victims; since then, this situation has changed substantially. Interest in gender issues in disaster research arose from the notion that a disaster is a physically and socially constructed event [2,3]. Natural hazards do not represent these types of events; rather, pre-existing social conditions are what determine whether an event is a disaster for the population [4]. In this sense, inequalities between women and men have a direct impact on the level of pre-disaster risk and on poverty and post-disaster exclusion [5,6,7,8]. Women have been found to be at greater risk than men of disasters, as evidenced not only by the marked difference in mortality rates between men and women but also in the access to and adequacy of care received, as well as those at risk of aggression [9,10,11,12,13]. The gender issue is a generic reflection of the society itself, and disaster events are the demonstration of that. Thus, it is mainly the male members who join the meetings/preparedness events, etc., and those who make decisions about prevention and intervention protocols, especially in more conservative and patriarchal societies. Therefore, gender must be considered when intervening in disaster situations, not only in the moments after it but in the preparation of the populations to face the risks [14,15,16,17].

### 1.2. Disasters, Gender, Health, and HIV

Disasters do not have the same consequences for the population as a whole, but social and economic inequalities make certain groups more vulnerable. The results derived from different studies show that women are more vulnerable in aspects related to education and work, are less likely to access resources and are more likely to need assistance from the state or non-governmental organizations after the disaster [4]. However, its negative consequences are not only limited to the economic sphere; they also affect the physical and mental health of those who experience them, particularly affecting women and limiting their life opportunities [5,18,19,20,21]. We know that although life expectancy is higher for women than for men, this difference does not translate to years of good health because, in fact, women face more years suffering from illnesses or disabilities [22]. We also know that the biological explanations for these differences are limited [23,24]. In recent decades, the prevalence and mortality of conditions such as lung cancer and cardiovascular disease have been increasing in women due to the progressive decrease in the differences in the consumption of alcohol and tobacco between men and women [25]. The objective of achieving greater gender equality in society has had the result of promoting in women the adoption of unhealthy practices initially considered masculine and, therefore, positively valued [26].

Gender inevitably intersects with factors that drive discrimination, marginalization, and social exclusion, with complex effects on the health and well-being of women [27]. Although progress has been made toward equality, inequalities in work, family responsibilities, and income have continued to exist, which have inevitably generated consistent differences between men and women in terms of health [28,29]. At the start of the 21st century, 60% of the people suffering from the human immunodeficiency virus (HIV) in underdeveloped countries were women, and, for the female population, this disease was the leading cause of death at reproductive age. Among the fundamental reasons for this change, the incidence of sexual assault and poverty has been pointed out [30,31,32,33].

The risk of becoming infected with HIV after a disaster is higher for women than for men. In addition, climate-related disasters worsen food security in less-developed countries, and they are strong predictors of the greater and disproportionate vulnerability of women to HIV [34,35]. Likewise, after a catastrophe, there is a decrease in the health of women because of the difficulties in accessing information and resources that make it possible for those women to take control of their own bodies [13,14,15,16,17,18,19,20,21,22,23,24,25,26,27,28,29,30,31,32,33,34,35,36].

### 1.3. The Haiti Earthquake

In 2010, an earthquake measuring 7.3 on the Richter scale struck one of the poorest countries in the world, Haiti, and caused one of the most significant human catastrophes in history. According to the United States Geological Survey (USGS), its epicenter was located approximately 25 km from Port-au-Prince, and the hypocenter was only 13 km from the surface, which caused the shock forces on the ground to be the most intense and destructive possible [37]. The earthquake killed at least 220,000 people and injured more than 300,000, nearly a million and a half people lost their homes, and the same number of people were displaced [38,39]. In a matter of seconds, the disaster wiped out 120% of the country’s GDP in 2009 [40]. In addition, it destroyed the country’s public health system, possibly the weakest in the hemisphere, and many clinics offering HIV testing, treatment, and care were devastated [41,42].

Currently, Haiti continues to suffer from the effects of the earthquake that struck the country in 2010. Considered the poorest territory in America, with a Human Development Index (HDI) in 2018 of 0.503 points, a life expectancy of 63.6 years, and a National Income Gross per capita of USD 1665 in 2017 [43], Haiti constitutes a territory of strong inequalities. If the previous indicators adjusted for inequality are considered, in 2018, the HDI suffered a decline of 40.5%, presenting an inequality in life expectancy of 32.2%, 37.3% in education, and 50.4% in income. With a Gender Inequality Index of 0.620, Haiti ranked 150th among the world’s countries ordered from lowest to highest regarding inequality between men and women in 2018, and it also had an adolescent fertility rate of 51.7, a gender differential in the percentage of the population with at least a second grade of education of 13 percentage points in favor of males, and an employment gap of 10 percentage points [43].

Regarding HIV, we should note that Haiti was among the first countries to report HIV infection registries, and it currently has one of the highest rates of HIV in Latin America and the Caribbean [44]. The data reported by the World Health Organization estimate that 37.9 million people were living with the human immunodeficiency virus worldwide at the end of 2019, of which approximately 330,000 people lived in the Caribbean, and of them, 160,000 people were Haitian. In that same year, the prevalence of HIV among Haitian adults between the ages of 15 and 49 was 1.9%; however, since 2000, it experienced continuous declines. The causes of these high levels of prevalence are manifold, but they fundamentally lie in the level of extreme poverty that affects a large part of the population, the scarcity of health resources, a very low educational context, risky behaviors, an increase in sex trafficking and prostitution, and very low levels of awareness about HIV and its transmission, among others [45,46]. The initial situation was already clear in terms of risks, as, according to data from the Demographic and Health Survey (DHS) on the 2010 Haiti earthquake, 14.5% of the women in the sample had already been raped by the age of 15 years, and more than three-quarters (79.8%) of the perpetrators of this sexual violence had been their current partners or husbands or one of their ex-partners. Women reported that their current partners had engaged in different types of sexual violence, such as physically forcing them to have unwanted sex (intercourse) and to perform other unwanted sexual acts [47]. The damage to health and the risks of seroprevalence are evident.

In summary, catastrophes or natural hazards situations affect men and women unequally, having more negative repercussions on women’s physical and mental health and leaving them in a situation of greater vulnerability. The aim of this study was to examine the impact the 2010 Haiti earthquake had on the seropositivity of female survivors. Using the differences-in-differences quasi-experimental technique, we assessed the impact of the earthquake on the gender relations associated with the probability of being seropositive in HIV, as well as the influence of associated variables that increase the penalties of the catastrophe linked to gender.

## 2. Materials and Methods

### 2.1. Sample and Procedure/Source of Information

The DHS, prepared by the United States Agency for International Development (USAID), constitutes the source of statistical information used. The DHS program was established in 1984, and, since then, surveys have been conducted in 85 countries; in 57 of them, at least two surveys were performed in successive waves. The surveys provide detailed and sex-disaggregated data on population, education, employment, health, gender roles, and living conditions within households. The sample design was carried out using group sampling stratified in two stages, and, in the case of Haiti, it contains representative information at the national level and by department.

Starting from 2001, the DHS survey was provided with a special module that collected the results of voluntary HIV tests on interviewees. The tests were carried out on men and women of reproductive age in an anonymous and informed manner, and these people were subjected to a high-level ethical review process [48]. Its link to the full DHS survey registry allows an in-depth analysis of the sociodemographic and behavioral factors associated with HIV infection.

The analysis carried out was based on the information provided by the surveys carried out in the 2005–2006 and 2012 waves, which provide data before and after the disaster and, consequently, allow an assessment of the impact. The HIV survey module contains information on 10,252 people surveyed in the 2005–2006 wave and 18,859 people for the 2012 wave. The analysis carried out did not take into account the observations that produced an indeterminate result in the HIV test. Therefore, the total of observations used was 29,083, of which 10,230 correspond to the 2005–2006 wave and 18,853 to the 2012 wave.

### 2.2. Method/Data Analysis

To measure the impact the earthquake had on gender relations associated with the probability of being HIV-positive, the differences-in-differences (DID) quasi-experimental technique was used, which uses longitudinal data from two population groups, namely treatment and control, to obtain an appropriate counterfactual that allows the estimation of a causal effect. This method of analysis has previously been used in different studies that have evaluated the impact of disasters on different aspects of health [49,50,51,52]. In our study, the DID estimator represented the difference between what happened before and after the 2010 Haiti earthquake between the group affected by the disaster and the control group [53,54].

Following Weitzman and Behrman (2016) [55], the application of DID was carried out by combining the information obtained from the DHS survey with the intensity of the earthquake to capture how it affected the results of the HIV test in the Haitian people interviewed and to analyze whether or not the probability of the positive result increased for women compared to men as a result of the disaster. Using a temporal variable, t, the observations related to the 2005–2006 wave constitute those not exposed to the disaster $\left(t=0\right)$, while those corresponding to 2012 were those related to the post-disaster $\left(t=1\right)$.

The 2012 DHS survey groups were assigned by mapping geocoded groups to the earthquake intensity map provided by the US Geological Survey (2010) [55] (Table 1). The intensity of the earthquake was measured from the department averages of the Mercalli (MMI). This scale assesses the effect of the earthquake on the earth’s surface, humans, objects in nature, and man-made structures on a 12-point ordinal scale, but not seismic risk. The MMI assesses the effect of the earthquake on the earth’s surface, humans, objects in nature, and man-made structures on a 12-point ordinal scale. The Mercalli scale considers an earthquake to be strong if it has an average score equal to or greater than 5 [56]. According to the departments for the 2010 Haiti earthquake, the average scores ranged from 4.7 in Nord (moderate) to 7.97 in Ouest (very strong), and these were included in two categories: “Moderate disaster” $\left(D=0\right)$, and departments with a mean Mercalli score higher than 5 as “Strong disaster”$\left(D=1\right)$.

As a dependent variable of the regression, we used HIV, a dichotomous variable that collects the result of the HIV serological status (1 for HIV positive and 0 for negative). According to the effects on the dependent variable from variable *S* (sex of the interviewee with a value of 1 for male and 0 for female) and its interaction with the temporal variable *t* and the variable *D*, we estimated the effect of the disaster on the gender relations associated with the probability of being seropositive for HIV.

Based on previous studies [31,45,57,58,59], we used control variables that were not correlated with the earthquake but that could explain the variations in HIV. In order to better estimate each person’s economic level’s effect on their HIV seropositivity, given the economic level’s high variability as a consequence of the disaster, we relied on previous studies that considered demographic variables such as the number of household members, their sex, and the age of the head of the household [14,60,61], as significant predictors of the wealth of individuals in poor countries, among others. The covariates considered were specified by: (1) age; (2) cohabitation status (1 if the person was married or living with a partner and 0 otherwise); (3) place of residence (1 if their residence was urban and 0 otherwise); (4) primary education (1 for yes and 0 for none); (5) higher education (1 for secondary or higher and 0 for none); (6) number of family members; (7) sex; (8) age of the person listed as head of the family.

The specification of the DID model is specified in:

(1) $$\begin{array}{l} P{\left(HIV\right)}_{i}=\beta_{0}+\beta_{1}\;D_{i}+\beta_{2}\;S_{i}+\beta_{3}\;t_{i}+\beta_{4}\left(D_{i}\times t_{i}\right)+\beta_{5}\left(S_{i}\times t_{i}\right)\;+\beta_{6}\left(D_{i}\times S_{i}\right)\;+\\ \;\;\;+\beta_{7}\left(D_{i}\times S_{i}\times t_{i}\right)\;+\cdots \;\;+\beta_{k}X_{ki}+u_{i} \end{array}$$

where *u* is the random disturbance term, and $X_{k}\;$ indicates each of the *k* covariates considered.

The DID was estimated from the interaction terms established between the different variables of the model. These terms collect the estimated differential effect with respect to the so-called base or control category, that is, the women surveyed who lived in the areas of Haiti that were only moderately affected by the earthquake.

Our objective in this work is to carry out an impact analysis, comparisons of effects between groups. For this reason, we use a DID model as the primary analysis. However, as the interaction effects that are the basis of the DID approach are not interpretable in nonlinear models such as probit or logit, the estimation of the model was carried out using a linear probability model [62,63], and its estimation was carried out by OLS. The LPM consistently estimates the conditional expectation of the result, has a simple interpretation and its use in a DID context is widely used [64,65,66]. The key parameter of interest in the DID describes is the parameter of the triple interaction term β7. This parameter measures the effect of the earthquake on the HIV gap between Haitian women and men. The statistical significance of the estimated coefficient of β7 shows us whether the disaster has an effect on the gender gap in HIV. The sign indicates the effect of the disaster on the gender gap in HIV. If it is negative, the gender gap will increase; if it is positive, the gender gap will decrease.

## 3. Results

The descriptive statistics corresponding to the selected variables are shown in Table 2. For the qualitative variables, information is displayed on the number of observations that satisfy the analyzed characteristics and their relative frequency. For the quantitative variables, the mean and the sample standard deviation are the indicators considered.

The HIV prevalence rate or HIV-positive percentage among surveyed adults who took the test remained stable. In the 2005–2006 wave, the prevalence rate among the population aged 15–49 was 2.2 (CI: 1.8–2.6), very similar to that relative to the wave of 2012, which was 2.2 (CI: 1.9–2.5).

**Table 2:** Descriptive statistics of the sample.

Variables	Categories	Moderate Disaster		Strong Disaster	
		2005–2006	2012	2005–2006	2012
**Dichotomic Variables**		**Frequency**	**Frequency**	**Frequency**	**Frequency**
HIV	Positive = 1	0.0232	0.024	0.022	0.022
Sex	Man = 1	0.483	0.501	0.481	0.497
Primary education	Primary education = 1	0.452	0.441	0.376	0.367
Secondary education	Secondary education or higher = 1	0.290	0.396 *	0.401	0.481 *
Status Cohabitation	In couple = 1	0.521	0.495	0.504	0.497
Head of household sex	Man = 1	0.594	0.611	0.595	0.619
Place of residence	Urban = 1	0.412	0.349 *	0.468	0.447 *
**Continuous Variables**		**Mean**	**Mean**	**Mean**	**Mean**
Age		290.578 (0.194)	290.465 (0.150)	290.214 (0.139)	290.496 (0.099)
Number of family members		60.315 (0.050)	60.310 (0.036)	50.667 (0.032)	50.456 (0.023)
Head of household age		470.258 (0.242)	470.652 (0.183)	440.762 (0.172)	440.617 (0.123)

* The contrast of the Student’s *t*-test for the comparison of the means and proportions for each of the waves in the survey by the territory of earthquake intensity is significant with *p* < 0.01. For continuous variables, the values in brackets correspond to the standard deviation. Sample sizes: moderate disaster zone (N2005 = 3616; N2012 = 6059); strong disaster zone (N2005 = 6614; N2012 = 12,794). Source: Own elaboration from DHS.

Regarding the sociodemographic characteristics of the people interviewed, the samples’ gender balance in both waves of the survey should be noted. In all regions and for the two waves of the survey, a high percentage of the population had a very low educational level. Of the total sample, more than 60% declared an educational level of primary school or lower, and only 3% of the people interviewed had a higher education level in 2005–2006, a figure that increased in 2012. The people surveyed averaged an age of 29 years and lived in households in which the head of the family was mainly male with an average age of 45 years, and they lived with more than five members in the household that was located mainly in urban areas.

Table 3 presents the results of the linear probability model on the probability of being seropositive. The temporary fixed effects on HIV prevalence are included in the parameter that accompanies the temporary variable t, and they were positive (0.015) and statistically significant (*p* = 0.0002), indicating a positive temporary fixed differential effect of 1.4% in the probability of HIV seropositivity.

The effect of the earthquake, regardless of sex, is reflected in the interaction term t×D and the coefficient β4 of the regression equation, which can be interpreted as the differences-in-differences term of the earthquake regardless of gender. Its sign and statistical significance showed a positive differential effect post-earthquake on the probability of contracting HIV, where the disaster increased the probability of being seropositive by one percentage point.

The sex variable, S, was incorporated into the model in additive and interaction terms. The β2 coefficient captured the fixed effect of sex on the probability of HIV. Their estimate indicated that the probability of HIV was reduced by 4.8 percentage points if the sex was male (−0.048; *p* = 0.000).

The estimate of the triple interaction term β7 was negative and statistically significant (−0.040; *p* = 0.008). The DID parameter, β7, measured the effect of the earthquake on the HIV gap between Haitian women and men. Its estimate indicated an increase in the probability differential of HIV between women and men of 4 percentage points as a consequence of the disaster.

Regarding the other covariates studied, the estimation of the coefficient associated with the age variable showed that an increase of one year in age increased the probability of contracting HIV by 0.216% (*p* = 0.004), which suggests that higher ages are associated with a greater risk of contracting HIV due to a longer risk period.

The results of the sign and statistical significance of the estimator of the higher education variable (−0.030; *p* = 0.012) showed that the attainment of higher education led to a lower risk of contracting HIV.

The characteristics related to the home of coexistence are important predictors of the probability of contracting HIV. Living with a partner reduced the probability of HIV incidence, and people who cohabited with a partner decreased the probability of HIV seropositivity by 3.3348% (−0.033; *p* = 0.000).

Taking into account the socioeconomic and productive structures of Haitian households, the variables number of family members, sex of the head of the family, and age of the head of the family are important predictors of their level of wealth, as we have previously described, and a positive association was obtained for this with the increase in the probability of HIV. Thus, as the number of people living in the household increased, the probability of HIV decreased (−0.002; *p* = 0.000), indicating a negative effect of this variable on the probability of being HIV-positive. An important result of our study, given its objective, was the estimated sign of the effect of the sex of the head of the family variable. If the household to which they belong was headed by a man, the probability of being seropositive reduced by 1.5264% (*p* = 0.107), and the age of the household leader was a significant predictor with a negative association (−0.000; *p* = 0.000). Finally, the sign of the place of residence variable showed a lower probability of seropositivity in residents of urban areas; however, this variable was not statistically significant.

**Table 3:** Differences-in-differences (DID) estimates of the effect of the earthquake on the probability of being seropositive.

Variable	Coefficient	Std. Error	t-Statistic	Prob.
C	0.068	0.032	2.119	0.034
t	0.014	0.003	3.761	0.000
D	−0.013	0.005	−2.681	0.007
S	−0.048	0.010	−4.682	0.000
t×D	0.010	0.003	2.969	0.003
t×S	0.022	0.006	3.303	0.001
D×S	0.035	0.017	2.024	0.043
t×D×S	−0.040	0.015	−2.635	0.008
Age	0.002	0.000	2.829	0.004
Primary Education	0.004	0.011	0.385	0.699
Higher Education	−0.030	0.012	−2.507	0.012
Cohabitation Status	−0.033	0.007	−4.594	0.000
Number of family members	−0.002	0.000	−3.690	0.000
Sex head of family	−0.015	0.009	−1.609	0.107
Age head of family	−0.000	0.000	−4.328	0.000
Place of residence	−0.004	0.003	−1.407	0.159

Note: Dependent variable, HIV. Method, a linear regression model with robust standard errors. The weights provided by the DHS survey were used. Sample sizes: moderate disaster zone (N2005 = 3616, N2012 = 6059); strong disaster zone (N2005 = 6614, N2012 = 12,794). Source: Our elaboration, based on the DHS. Std. Error, standard deviation; Prob., *p*-value.

### Robustness Tests

Before estimating the model, we have made the contrast of key assumptions in the DID modeling. The first of these is the so-called “assumption of common trends” or “parallel trends” [67,68], which indicates that the indicators of interest follow the same temporal trajectory in the treatment and comparison groups. If this assumption is not verified, the estimates made through this methodology are not valid since the effect of the earthquake will be confused with the difference in trends. Although it is not possible to test this assumption, since we cannot observe the affected group in the absence of the earthquake, we can obtain some indication of its validity. As we did not have information prior to the wave of the 2005−2006 survey, we opted for the alternative of analyzing the assumption by running a “placebo” test [67,68]. We used two placebo tests, one relative to the group affected by the earthquake and the other relative to the control group.

Table 4 shows the placebo test results in the control group. Differences in differences are estimated with a “false” treatment group, in our case, departments that have been moderately affected by the earthquake, and the result of the impact should be null. To do this, we divided the control group D=0 randomly, and we consider that the North and North-East regions were strongly affected by the *D* = 1 earthquake. The results show that the estimated coefficients are not significant at 95% confidence, which indicates that the parallel trends between both groups are maintained in the two periods of time for the result variable. In this way, the assumption of parallel trends is validated.

In a similar way, we carried out the placebo test in the treatment group (Table 5). In this case, we only considered the departments with an earthquake intensity greater than 5, D=1. We divide this sample into two subsamples, a control group for which we consider the departments of Sud, Center, and Ouest that will constitute the untreated group, D=0, and the departments of Artibonite, Nippes, and Sud-Est that will be affected by the earthquake. The results of the differences-in-differences model again show non-significant estimators, which indicates the validity of the assumption of parallel trends.

**Table 4:** The validation of parallel trends with the placebo test control group.

Variable	Coefficient	Std. Error	t-Statistic	Prob.
C	0.038	0.009	3.893	0.001
t	−0.001	0.008	−0.028	0.978
D	0.003	0.007	0.545	0.586
S	−0.009	0.007	−1.261	0.207
t×D	0.005	0.009	0.535	0.593
t×S	−0.006	0.011	−0.571	0.568
D×S	0.007	0.010	0.743	0.457
t×D×S	0.001	0.013	0.110	0.912
Age	0.001	0.001	2.925	0.003
Primary Education	−0.005	0.004	−1.314	0.189
Higher Education	−0.012	0.005	−2.440	0.015
Cohabitation Status	0.003	0.004	0.939	0.348
Number of family members	−0.001	0.000	−2.686	0.007
Sex head of family	−0.006	0.003	−2.045	0.041
Age head of family	−0.001	0.001	−2.464	0.014
Place of residence	0.002	0.004	0.746	0.456

Note: Dependent variable, HIV. Method, a linear regression model with robust standard errors. The weights provided by the DHS survey were used. Sample size: 9675. Source: Our elaboration, based on the DHS. Std. Error, standard deviation; Prob., *p*-value.

Given that the DID estimation assumes the absence of contamination in the comparison groups, the migrations could have biased our results. In order to validate the proposed model, we estimated it only for those households that, in 2012, remained in the same residence as before the earthquake (wave of the 2005–2006 survey). The results obtained (Table 6) confirmed our analysis, as the sign and statistical significance of the variables that were considered remained stable in relation to those that were obtained in the original regression.

On the contrary, it was necessary to analyze the possible variations in the control variables over time between the treatment and control groups that might have contaminated the effect of the earthquake on the prevalence of HIV. The results of the comparison contrasts of the means and proportions (Table 1) suggested that the sample means were stable; however, the null hypothesis was not maintained for the variables related to higher education and place of residence. This fact may be caused by mortality or migration as a consequence of the disaster; therefore, it was necessary to carry out a validation of the model, taking into account only those households that remained in their place of residence during the period considered. The results obtained (Table 4) were substantially similar to those of the main regression, which supports our analysis.

## 4. Discussion

The analyses that were carried out based on the data from the DHS survey on the Haiti earthquake, through the implementation of the DID method, made it possible to establish some relevant conclusions regarding the gender gap associated with the disaster. Among the main points to highlight is that the Haiti disaster showed gender biases in its impact on the seropositivity rate.

This study found that the earthquake increased the probability of contracting HIV for the entire population, which is why it is a health issue that would require immediate attention in terms of the global action of the institutions interested in caring for victims of disasters. However, the effects of the catastrophe were not the same regardless of the gender of the people affected: a seropositivity differential of four points was observed between women and men after the earthquake. The signs and statistical significance of the covariates considered were consistent with other empirical research on contextual factors associated with HIV in developing countries [31,58,69,70,71,72].

The study controlled for the effects of different covariates that have made it possible to provide an image of the effects of the earthquake according to different characteristics of women’s lives. Thus, our results indicate that older ages are associated with a higher risk of HIV infection due to a longer risk period [45,58]. As Austin et al. (2020) [35] pointed out regarding climate disasters, they are a significant factor shaping women’s HIV vulnerability indirectly through increased food insecurity and resource deprivation. Both processes alter social relationships and behaviors, including risky sexual behaviors, forced sex, and transactional sexual relationships, which serve to escalate HIV transmission among vulnerable women in poor countries. According to our results, permanence over time in discriminatory situations with scarce resources and means both to treat and prevent the disease implies an increased risk of the prevalence of HIV for older women.

Regarding the educational level achieved, it was observed, as in other international studies [45,73], that higher educational levels are associated with a lower risk of HIV infection. This is consistent with reports from international organizations that indicate that educational level improves the ability to understand and act on health promotion messages, as well as greater exposure to school HIV prevention programs or greater access to health services [74]. The educational level allows an individual or group of people to gain a clear perspective on the problems of life, including the prevention of diseases and, therefore, HIV. In the sample from Haiti, the pre- and post-earthquake academic training of women was lower than that of men.

On the contrary, it has also been found that certain characteristics of coexistence are good indicators of the probability of contracting HIV. Thus, living with a partner decreases the probability of HIV incidence, and this result is consistent with previous studies that showed how living with a partner decreases the probability of HIV [45,58,75]. Undoubtedly, this does not mean that any type of partner can be associated with a decrease in the probability of contracting HIV, as those in which sexual violence has increased [47] could promote it.

Poverty and the scarcity of resources may be related to these results due to their unequal distribution according to whether one is male or female. The wealth variable is an important predictor of the probability of HIV [31,57,58]. However, the DID analysis does not allow us to consider this variable in the model, as it would skew the results as it experienced significant variations as a consequence of the disaster. Therefore, other variables that characterize the household were used, which are significant predictors of its wealth but are invariant. In fact, in poor countries, different studies considered demographic variables such as the number of its members, sex of the head of the household, age of the head of the household, ethnicity, and religion, among others [14,60,61], as predictors of the level of household wealth, among others. In our analysis, we considered the following as variables that, without being directly and significantly affected by the disaster, characterize the household and provide an approximation to its level of wealth: the number of family members and the sex and age of the head of the household. In fact, the productive structure of Haiti is marked by informal jobs making up 80% of employment, a high polarization toward the primary and tertiary sectors, and 40% of children between 7 and 14 years of age performing paid activities [76], making the number of members that make up the family unit an important predictor of household wealth. In addition, different studies have shown a positive effect of the age and sex of the head of the household on the wealth level of Haitian households that are richer when the head of the household is male and older [77,78,79].

The results of this study on these issues are also interesting, as they point out that as the number of people living in the home increases, the probability of HIV decreases, indicating a negative effect of this variable on the probability of being HIV-positive. Furthermore, households headed by women are more likely to be affected by HIV (*p* = 0.107), and this is a fact that necessarily has political implications regarding the interventions of international organizations involved in protecting health, as well as for those who seek to promote equality between men and women. This is a result that supports the growing research on increased post-disaster social vulnerability for female-headed households [9,80]. Arenas (2001) [80] evaluated the socioeconomic damage of earthquakes in El Salvador in the informal economy of women. They observed how disasters increase their vulnerability, as well as their families’, through the reduction in their formal and informal work. Furthermore, the fact that women extend their reproductive roles from family to community with unpaid and nonbasic decision-making tasks further increases their levels of vulnerability [81]. Bradshaw and Arenas (2004) [82] found different sources of vulnerability in families headed by women during and after Hurricane Mitch: a greater dedication to reproductive and community work, loss of a regular source of income, an increase in the households headed by women and single mothers, violence against women, etc. In Haiti, it has also been shown that the gender gap in social vulnerability increased for households headed by women [14]. The results derived from our research reaffirm those obtained in different studies that show how disasters do not produce equal consequences for the population as a whole but that social and economic inequalities make certain groups more vulnerable, even to HIV. Lastly, our analysis did not find a significant association between living in urban settings and the probability of HIV, possibly because of the particular characteristics of the Haitian disaster, which mainly affected urban areas.

The main methodological problem that we considered as associated with the DID analysis carried out is a consequence of the particular situations experienced post-disaster. The earthquake left more than 1.5 million people homeless, and many of them were forced to move into camps in Port-au-Prince and surrounding areas, where many women turned to formal and informal sex work to survive. A study carried out by the United Nations Population Fund revealed that five months after the earthquake, more than 10% of women living in camps were pregnant, and the majority of these pregnancies were unwanted [83]. The number of women who suffered rape, gang rape, sexual assault, intentionally harmful assault, unwanted physical contact, child sexual abuse, and intimate partner violence increased as a result of the disaster [38,47,84]. The DID estimation assumes that the absence of contamination in the comparison groups and migrations could have biased our results. Despite knowing that a characteristic of these migrations was that more than 70% of the displaced people stayed close to their places of origin or returned to them after a year, and foreign migration was very limited [85], new analyses were carried out for households that, in 2012, remained in the same residence as before the earthquake. The results again validated those obtained in the original regression, thus reinforcing the conclusions reached with the larger data analysis.

The possible downward bias of our results should also be noted. At the end of 2010, a cholera epidemic caused the mortality of at least 8000 people, and the effects of disasters were not gender-neutral. Different investigations that were carried out indicated that disasters do not affect the entire population in the same way but that the most vulnerable sectors—and among them, women—are the hardest hit by the consequences of disasters [86,87,88,89]. It is very likely that the effects of the cholera epidemic disproportionately affected the most vulnerable sectors of the Haitian population—poor women, among others—which would produce a sample bias by not considering this population in the sample.

Therefore, there were limitations to the presented study, which requires future clarification, as investigating the relationship between poverty and disaster risk is a complex task, particularly in low-income territories where the scarcity of data is a common characteristic.

First, it should be noted that the results of this study are limited by information from DHS. In this sense, the consideration of other aspects related to gender in the Haitian context would probably have shed more light on our work. In methodological terms, it is necessary to indicate that we have used as a measure of the disaster only the seismic intensity and not the seismic risk. We are not aware of any work that measured the average seismic risk by department in the 2010 Haiti disaster, which leads us to judge the disaster based on the intensity of the earthquake. It should be noted that the MMI is a purely hazard-related measure.

As we said before, gender intersects with factors that drive discrimination, marginalization, and social exclusion, with complex effects on the health and well-being of women [27], not only about seropositivity. Gender inequality transforms into health risks through discriminatory values, norms, beliefs, and practices, as well as differential exposures and susceptibilities to disease, disability, and injury, and biases in health systems and health research. This is the case of the differential exposure to HIV after the earthquake in Haiti. Not taking gender issues into account in the planning of any health intervention would be a serious error, as the results show that the increase in seropositivity in women was 4.8 percentage points in the post-disaster period. Previous studies had shown that after disasters and during humanitarian crises, guaranteeing continued access to infection prevention methods and access to treatment programs is a priority to guarantee the sexual and reproductive health of the community [51,90]. The absence of health and social resources leaves women at a clear risk of contracting HIV in their search for basic survival resources for themselves and their children. Advancing care and assistance to female victims of disasters will only be possible through approaches that advocate for equality between women and men in terms of access to and fair distribution of opportunities, rights, obligations, and resources [91]. This research shows that Haitian women had an increase in seropositivity not due to chance and that when their family depended on them, the risk was even greater: the earthquake and HIV are not gender-neutral.

As we are now in the second decade of the 21st century, it is necessary to incorporate gender considerations into healthcare, but despite evidence of inequalities demonstrated by research, actions in this regard have been insufficient. The reviews carried out show how publications on how to address gender inequalities in health have increased, but good intentions have not been sufficiently realized.

## 5. Conclusions

After a disaster, the population experiences both negative economic consequences and negative impacts on their physical and mental health, limiting their life opportunities. Faced with a catastrophic situation, there is an increased vulnerability associated with gender as poverty and exclusion situations rise in the female population compared to the male, which generates an increase in the gender gap that is also a consequence of the catastrophe.

Gender inevitably intersects with factors that drive discrimination, marginalization and social exclusion with complex effects on the health and well-being of women. The risk of becoming infected with HIV after a disaster is higher for women than for men.

Based on the analysis of the catastrophe that occurred in Haiti in 2010, we have analyzed the impact of the earthquake on gender relations associated with the probability of being HIV-positive, using the quasi-experimental technique called differences in differences. From their results, we can extract, among other things, that the catastrophe is not indifferent to gender, since as a consequence of the disaster, the estimate indicates an increase in the probability differential of HIV between women and men of 4 percentage points, favoring men. Additionally, the results indicate that when a man shares the household, the probability of being zero positive is reduced. In summary, this highlights a profile of women more susceptible to zero positivity and a situation of vulnerability: old women with low educational levels who live alone or are the only source of income for the household.

## Figures and Tables

**Table 1:** Average scores of the Mercalli earthquake Haiti 2010 by department.

Department	Mean Mercalli Score	Standard Deviation
Nord	4.70	0.55
Grand’anse	4.71	0.14
Nord-Est	4.79	0.05
Nord-Ouest	4.80	0.30
Artibonite	5.17	0.33
Sud	5.32	0.66
Centre	5.33	0.33
Nippes	5.60	2.26
Sud-Est	6.44	1.86
Ouest	7.97	1.42

Source: Data from [55].

**Table 5:** The validation of parallel trends with the placebo test treatment group.

Variable	Coefficient	Std. Error	t-Statistic	Prob.
C	0.013	0.007	1.987	0.047
t	0.003	0.004	0.720	0.471
D	0.001	0.005	0.203	0.839
S	−0.005	0.005	−1.140	0.254
t×D	−0.002	0.007	−0.325	0.745
t×S	−0.003	0.006	−0.597	0.551
D×S	0.001	0.008	−0.025	0.980
t×D×S	0.001	0.009	0.062	0.950
Age	0.001	0.000	7.535	0.000
Primary Education	0.006	0.003	1.960	0.050
Higher Education	−0.003	0.003	−0.919	0.358
Cohabitation Status	0.001	0.003	0.288	0.773
Number of family members	−0.002	0.000	−5.296	0.001
Sex head of family	−0.008	0.002	−3.386	0.001
Age head of family	0.000	0.000	−1.197	0.231
Place of residence	0.008	0.002	3.461	0.001

Note: Dependent variable, HIV. Method, a linear regression model with robust standard errors. The weights provided by the DHS survey were used. Sample size: 19,408. Source: Our elaboration, based on the DHS. Std. Error, standard deviation; Prob., *p*-value.

**Table 6:** Validation assuming the absence of contamination.

Variable	Coefficient	Std. Error	t-Statistic	Prob.
C	0.019	0.007	2.420	0.015
t	0.000	0.004	0.070	0.944
D	−0.003	0.003	−0.958	0.338
S	−0.003	0.003	−0.969	0.332
t×D	0.009	0.006	1.381	0.167
t×S	−0.004	0.005	−0.713	0.475
D×S	0.001	0.005	0.287	0.773
t×D×S	−0.016	0.005	−3.248	0.001
Age	0.000	0.000	5.057	0.000
Primary Education	0.002	0.004	0.517	0.604
Higher Education	−0.004	0.004	−0.996	0.318
Cohabitation Status	0.000	0.003	0.243	0.807
Number of family members	−0.001	0.000	−4.743	0.000
Sex head of family	−0.001	0.002	−0.437	0.661
Age head of family	−0.000	0.000	−1.569	0.116
Place of residence	0.004	0.002	1.600	0.109

Note: Dependent variable, HIV. Method, a linear regression model with robust standard errors. The weights provided by the DHS survey were used. *N* = 13,156. Source: Our elaboration, based on the DHS. Std. Error, standard deviation; Prob., *p*-value.

## Data Availability

The data that support the findings of this study are available from the corresponding author, upon reasonable request and with the approval of DHS.
